# Indoor Secondary Pollutants Cannot Be Ignored: Third-Hand Smoke

**DOI:** 10.3390/toxics10070363

**Published:** 2022-06-30

**Authors:** Jia-Xun Wu, Andy T. Y. Lau, Yan-Ming Xu

**Affiliations:** Laboratory of Cancer Biology and Epigenetics, Department of Cell Biology and Genetics, Shantou University Medical College, Shantou 515041, China; 20jxwu2@stu.edu.cn (J.-X.W.); andytylau@stu.edu.cn (A.T.Y.L.)

**Keywords:** indoor secondary pollutants, third-hand smoke, TSNAs, NNA, NNN, NNK, PAHs

## Abstract

Smoking has been recognized by the World Health Organization (WHO) as the fifth highest threat to humanity. Smoking, a leading disease promoter, is a major risk factor for non-communicable diseases (NCDs) such as cancer, cardiovascular disease, diabetes, and chronic respiratory diseases. NCDs account for 63% of all deaths worldwide. Passive smoking is also a health risk. Globally, more than a third of all people are regularly exposed to harmful smoke. Air pollution is a common global problem in which pollutants emitted into the atmosphere undergo a series of physical or chemical reactions to produce various oxidation products, which are often referred to as secondary pollutants. Secondary pollutants include ozone (O_3_), sulfur trioxide (SO_3_), nitrogen dioxide (NO_2_), and respirable particulate matter (PM). It is worth mentioning that third-hand smoke (THS), formed by the reaction of nicotine with second-hand smoke (SHS) caused by indoor O_3_ or nitrous acid (HONO), is a major indoor secondary pollutant that cannot be ignored. As a form of indoor air pollution that is relatively difficult to avoid, THS exists in any corner of the environment where smokers live. In this paper, we summarize the important research progress on the main components, detection, and toxicity of THS and look forward to future research directions. Scientific understanding of THS and its hazards will facilitate smoking bans in indoor and public places and raise public concern for how to prevent and remove THS.

## 1. Introduction

Tobacco use causes 20% of cancer deaths worldwide, and it is estimated that tobacco-related deaths will result in 10 million deaths annually. Tobacco is one of the leading preventable causes of illness and death around the world [1,2]. Environmental tobacco smoke (ETS) is a significant source of particles and gases in the indoor environment. Exposure of tobacco smoke to O_3_ may contribute to the formation of ultrafine particles (UFP) in the indoor environment [3]. In addition to the common SHS, THS is also an important part of non-smokers’ exposure to tobacco smoke components. THS is a secondary pollutant formed by the reaction of adsorbed nicotine with indoor O_3_. The concept of THS was first introduced in 2009 by doctors at Massachusetts Children’s General Hospital in Boston [4]. THS refers to the contamination that remains on the surface of objects after the SHS has been cleared. In other words, first-hand smoke is smoke that the smoker inhales into his or her lungs. SHS is the mixture of mainstream smoke exhaled by the smoker with other substances produced by sidestream smoke from burning cigarettes that enter the air and maybe inhaled or ingested into the lungs and stomach to be absorbed or digested. THS refers to smoke pollutants that remain on surfaces such as clothing, walls, furniture, hair, skin, carpets, and dust after smoking. These adhering pollutants can be re-emitted into the gas phase or react with oxidants and other pollutants commonly found in the indoor environment to form secondary pollutants, including carcinogenic compounds [4].

THS is a secondary toxicant, a type of secondary aerosol. THS has characteristics that can be described by “the four Rs” because it (1) remains as a residue on the smoker’s body and in the places where they have been smoking; (2) reacts with chemicals in the air to generate more toxic chemicals; (3) re-emits, that is, the generation of these toxic chemicals can be re-released into the air; (4) and can be resuspended long after smoking has ended to enter the body through inhalation, from hand to mouth, and skin absorption, resulting in a lasting, serious impact on human health. Therefore, it poses a potential health hazard to both smokers and non-smokers.

Many studies have proven that SHS is absolutely no less harmful than direct smoking. Centers for Disease Control and Prevention (CDC) data showed that SHS exposure increases the risk of heart disease, lung cancer, and stroke in non-smokers [5] and the number of deaths caused by SHS exposure in China exceeds 100,000 [6]. However, when SHS dissipates, it contains harmful chemicals that can be deposited on surfaces over time, and this accumulated chemical residue constitutes THS. THS as indoor pollution is long-term and cumulative, and its toxicity will gradually increase with the passage of time. Moreover, the main route of exposure to SHS is inhalation, and its exposure time is short. In contrast, THS is not only inhaled, but can also be absorbed through skin contact with contaminated surfaces, possibly including the smoker’s clothing, and their body parts. This illustrates that THS is one of the major indoor secondary pollutants with the same dangers and potential to harm human health as SHS. Thus, THS is a unique entity that constitutes a risk to the health of children and adults, and study of the concept is necessary.

Cigarette smoke itself is a complex aerosol consisting of gases and volatile chemicals with suspended PM. SHS contains the same harmful chemicals, including some carcinogens, which are found in the smoke inhaled by smokers. The gas-phase includes neurotoxic nicotine, carbonyl compounds (such as acetaldehyde, formaldehyde, and acrolein), hydrocarbons (such as benzene, toluene, and some polycyclic aromatic hydrocarbons [PAHs]), nitrogen oxides (NO), pyridine, ammonia, nitrosamines, hydrogen cyanide, and 1,3-butadiene, among other harmful gases. The particulate phase is often referred to as the “tar” component, which contains benzene, tobacco-specific nitrosamines (TSNAs), benzo[α]pyrene (BaP), cadmium (a toxic metal), formaldehyde and acetaldehyde, etc. These components are irritating to the eyes and respiratory tract, as well as hazardous to the cardiovascular and reproductive systems [7]. Compared to directly inhaled tobacco, SHS contains 5 times more carbon monoxide (CO), 3 times more nicotine, 4 times more BaP, and 50 times more nitrosamines than mainstream smoke. THS is created at the same time that SHS is created, and after the gas-phase component of SHS is removed with time and air movement, THS smoke becomes the primary exposure, and studies have shown that the toxicity of THS increases over time. Nicotine in THS can react with HONO in the air to produce carcinogenic nitrosamines, which results in a much higher nitrosamine content in THS than in SHS. Some studies have pointed out that the ratio of 4-(methyl nitrosamine)-1-(3-pyridyl)-1-butanone (NNK): nicotine on cotton exposed to smoke is 10 times higher than that in aerosol samples, which means that the carcinogenic NNK content in THS is higher compared to SHS [8].

## 2. Components in THS

Cigarette smoke contains a complex mixture of particles and gases, including nicotine, carbon monoxide, nitrogen oxides, different functional and volatile organic compounds, and metals, all of which are major sources of pollution in the indoor environment. Nicotine is released and deposited almost completely on indoor surfaces, where it can react with O_3_, HONO, and other atmospheric oxidants to produce carcinogenic TSNAs [9]. To date, many harmful components have been detected in THS, including those specific to tobacco combustion, such as nicotine and TSNAs, as well as tobacco-related toxicants, N-nitrosamines, aromatic amines, PAHs, and volatile carbonyls [8,10,11,12,13]. Among the components that are most harmful after long-term exposure through inhalation are PM2.5, acrolein, furans, acrylonitrile, 1,3-butadiene, acetaldehyde, isoprene, toluene, and benzene, and these persist long after smoking has ended [8]. SHS emissions can be divided into the following categories according to vapor pressure range (Table 1): gaseous inorganic compounds (>13 kPa), very volatile organic compounds (VVOCs) (>7 to 13 kPa), volatile organic compounds (VOCs) (0.01 to 10 kPa), semi-volatile organic compounds (SVOCs) (10^−2^ to 10^−8^ kPa), and particulate organic compounds (<10^−8^ kPa) [14]. Components released from SHS lead to the formation of THS through dynamic processes, such as deposition, adsorption and desorption, and chemical reactions, which changes the chemical composition and concentration of components of environmental tobacco smoke in the indoor environment.

Extremely VOCs are unlikely to remain on surfaces and, in general, they are not very well adsorbed on indoor surfaces and can be removed by ventilation. Therefore, the gaseous inorganic compounds in THS are very small. In contrast, VOCs are more likely to be adsorbed to the surface of objects, but it is not possible to determine whether the identified VOCs are secondary pollutants because it is not clear whether they are the result of direct combustion during smoking or a chemical reaction between products during combustion. SVOCs are present in the indoor environment as gases, liquids, and solids and form partitions between indoor air and surfaces. SVOCs include alkanes (C_16_–C_22_), PAHs, quinolines, isoquinolines, carbazoles, and nicotine (such as N-nitrosonicotine (NNN) and NNK). Smoking-induced combustion forces SVOCs into the gas phase. As they cool, they condense into liquid droplets and adsorb to particles and chamber surfaces. Once adsorbed, they can also be re-emitted into the gas phase. The tendency of SVOCs to attach to dust and surfaces reduces the likelihood of their removal by ventilation and increases the likelihood that they will remain indoors long after active smoking has stopped, which may be the main form of THS. The exposure of cigarette smoke to O_3_ may lead to the formation of ultrafine particles in the indoor environment. The growth of primary particles and the formation of ultrafine particles are caused by the rapid reaction between the gas phase substances in cigarette smoke and O_3_ [3]. Emission and ventilation rates typically have the greatest impact on indoor concentrations of PM, with small amounts of PM also deposited on indoor surfaces. Over time, as gas-phase SVOCs are removed from the air and deposited onto surfaces, there may also be a loss of SVOCs from PM. As gas-phase SVOCs are deposited onto surfaces, particle-phase SVOCs are volatilized from the particles to restore the equilibrium between the two phases [15]. Therefore, PM such as BaP, benzo[β]fluoranthene, benzo[κ]fluoranthene, and solanesol are also present in THS.

The formation of secondary organic aerosol (SOA) and carcinogens is an important aspect of the conversion of SHS into THS. This process includes the secondary reaction of SHS with O_3_, the reaction of nicotine with OH, the multiphase oxidation of nicotine and cigarette smoke on indoor surfaces to form SOA, and the reaction of HONO with surface-adsorbed nicotine to form carcinogenic TSNAs. The following section will describe the toxicity of tobacco-specific nitrosamines and polycyclic aromatic hydrocarbons, the major carcinogenic components of THS.

## 3. Generation of THS

Studies have shown that THS can be generated by several physical and chemical transformation mechanisms, as follows:

- Selective adsorption and accumulation of certain compounds, such as SVOCs, from the gas phase of SHS onto the surface of objects before they are slowly released into the air;
- PM from SHS stains furniture, walls, carpets, clothing, etc., which can then again be suspended into the air;
- Certain compounds in SHS, such as nicotine, attach to the surface of objects and react with O_3_, HONO, and other substances in the atmosphere to form second-generation toxicants.

## 4. Places Where THS Exists

As described before, THS is one of the main indoor secondary pollutants and is commonly found in smokers’ homes. However, in countries where smoking bans are not strictly enforced in public places, exposure to SHS is higher in public places than in homes, which leads to a high presence of THS in these places as well. THS is also common in car indoor environments. Due to the small space inside a car, and an enclosed environment with a higher surface-to-space ratio, they are more likely to absorb the toxicants in smoke. High concentrations of nicotine and TSNAs are found wherever a smoker has been, and as a result of human activity and air circulation, THS is everywhere, and traces can even be found in non-smokers’ homes and non-smoking areas.

## 5. The Main Carcinogenic Components of THS

**Figure 1:**
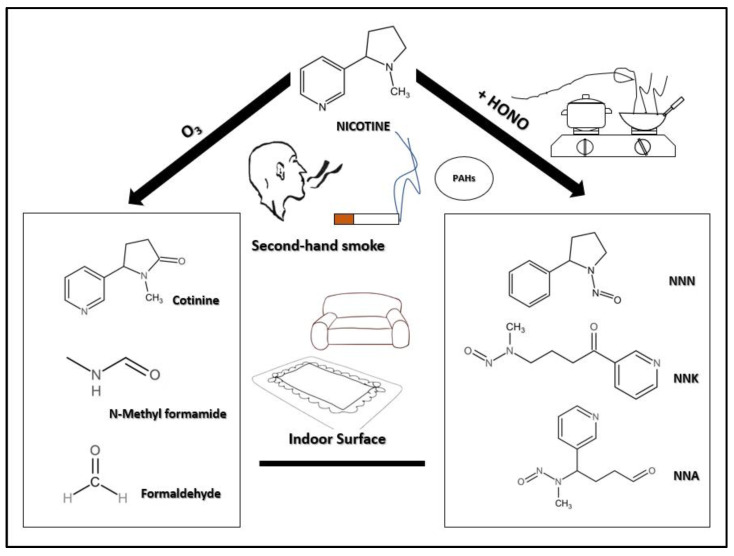
The focus of THS generation is on chemical transformations related to nicotine. There are two main categories: one is the chemical transformation of nicotine and O_3_, forming SOAs and some oxidation products. The other category comprises TSNAs that can be formed by the reaction of nicotine with HONO.

The main chemical components of THS include nicotine, cotinine, 3-vinyl pyridine, cresol, naphthalene, PAHs, formaldehyde, TSNAs, and respirable volatile organic compounds including acetonitrile, benzene, acetone, and toluene [8,9,10,16]. The focus of THS generation is on nicotine-related chemical transformations, which fall into two main categories: First, the chemical transformation of nicotine and O_3_ to form an SOA and some oxidation products. In many places, O_3_ is used to remove the smell of smoke, which can potentially cause the production of THS and increase secondary pollution; therefore, it plays an important part in the production of THS. Second, TSNAs can be formed by the reaction of nicotine with HONO (Figure 1). To better understand the potential health hazards of THS, we will examine its main constituents, PAHs and TSNAs, and their respective molecular effects that induce cancer.

### 5.1. PAHs: The Killer in Cigarette Smoke

Tobacco smoke is one of the major sources of PAHs in settled house dust (SHD), and PAHs from tobacco smoke is a component of THS [11]. Studies have shown that SHD in smokers’ homes contains higher levels of PAHs than SHD from non-smokers’ homes. BaP, one of many polycyclic aromatic hydrocarbon carcinogens in tobacco smoke, includes BaP, methyl isobutenate, dibenzo[a, h]anthracene, etc. [17]. In recent years, many types of research have investigated the toxicity genomics of PAHs, which can be divided into the following four groups:

(1) PAHs can cause DNA damage by producing DNA adducts. PAHs mediating DNA adduct formation is an indicator of DNA damage and a biomarker of potential cancer risk. Upon entry into the body, PAHs firstly mediate the activation of cytochrome P450 (CYP), which induces glutathione S-transferase, UDP glucuronosyltransferase, etc., via aromatic hydrocarbon receptor-dependent or independent pathways. PAHs are metabolized by the CYP1A1/1B1/EH pathway, CYPase pathway, and AKR pathway to form free radical cations and quinones, which are carcinogenic substances and produce DNA adducts causing DNA mutations, thus leading to cancer development. BaP is a potent lung carcinogen that is slowly absorbed by tracheal epithelial cells and induces mainly squamous cell carcinoma [18,19,20]. BaP-DNA adducts may cause mutations in the p53 gene [21,22]. BaP acts as a ligand for AhR and activation of AhR has a variety of downstream effects, including the formation of DNA adducts (via CYP1A/1B1-dependent metabolic activation), tumorigenesis, inflammation, cell proliferation, and loss of cell adhesion. BaP is capable of causing p53 mutant overexpression with DNA damage, resulting in cervical tissue damage. The expression levels of Bcl-2, Bax, Caspase-3, Ki-67, and C-myc are also significantly altered in mouse cervical tissue cells treated with BaP, or in mouse cerebral cortex and hippocampus regions, so BaP affects the expression of genes associated with apoptosis and triggers cervical cancer or chronic neurotoxicity [23,24,25]. In the lung tissue of smokers, CYP1-mediated oxidation and bioactivation of BaP leads to the formation of benzo[α]pyrene-7,8-diol-9,10-epoxide, which results in the formation of DNA adducts, an established risk factor for lung tumors [26].

(2) PAHs can regulate gene polymorphisms and alter epigenetic patterns, such as CYP1A1, CYP2E1, GSTM1, GSTTQ, GSTT2, AhRA and other gene polymorphisms [27,28,29]. It has been reported that PAHs may produce insulin resistance through methylation-mediated inhibition of normal IRS2 gene expression and cause lipid metabolic syndrome [30].

(3) Inhalation of PAHs causes oxidative stress and inflammation in the body by inducing reactive oxygen species (ROS) production in cells [31]. Mitochondria are the main site of ROS production, and BaP induces excess free radical production in mitochondria [32]. It was also found that BaP treatment increased malondialdehyde and glutathione levels and significantly increased the activities of antioxidant enzymes such as superoxide dismutase (SOD), glutathione peroxidase (GPx), and catalase (CAT) in mice, suggesting that the addition of BaP may cause oxidative stress and oxidative DNA damage [33].

(4) Alteration of self-physicochemical properties and disruption of internal environmental homeostasis leads to the production of procarcinogens. Prolonged exposure to PAHs, the precursor compounds of PAHs, or their metabolites, in the body can perturb the intracellular environment and be toxic or even carcinogenic to tissues [34]. Exposure to PAHs in cigarette smoke allows substantial replication of the HIV virus in macrophages from HIV-positive smokers, possibly because the expression of CYPs is regulated by aromatic hydrocarbon receptors that allow PAHs to mediate the production of procarcinogens or toxic metabolites [35].

### 5.2. TSNAs: Products of the Reaction between Nicotine and HONO

Residual nicotine from tobacco smoke is adsorbed to indoor surfaces and reacts with environmental HONO to form carcinogenic TSNAs, which are among the most widespread and potent carcinogens present in unburned tobacco and tobacco smoke [36,37]. Indoor HONO and its precursors NO and NO_2_ come not only from smoking but mostly from indoor combustion sources, such as improperly ventilated gas stoves and heaters, as well as from outdoor air pollution infiltration generated by vehicle exhaust or biomass combustion [38]. Common TSNAs include NNK, NNN, 4-(methylnitrosamino)-4-(3-pyridyl)-1-butanol (iso-NNAL), N-nitrosoanabasine (NAB), and N’-nitrosoanatabine (NAT). Three major nitrosamines are formed in the reaction of adsorbed nicotine and gaseous HONO: (i) NNK, nicotine-derived nitrosamine ketone, specifically 4-(methylnitrosamido)-1-(3-pyridyl)-1-butanone; (ii) NNA, nicotine-derived nitrosaminoaldehyde, mainly 1-(N-methyl-N-nitrosamines)-1-(3-pyridyl)-4-butanal; and (iii) NNN, N’-nitrosonornicotine. NNN, NNA, and NNK are the most carcinogenic components of TSNAs [39,40,41,42,43]. Nitrosamines require metabolic activation to bind to DNA and other cellular macromolecules to exert their effects.

NNK is a highly carcinogenic TSNA that can be formed when cigarette sidestream smoke is released into the ambient air. NNK and its metabolic breakdown product, 4-(methylnitrosamino)-1-(3-pyridyl)-1-butanol (NNAL), are the most potent carcinogens among TSNAs [39]. Naturally occurring NNK in tobacco smoke is inert and requires activation of multiple CYPs as DNA-reactive metabolites. Activated NNK induces metabolic activation of α-hydroxylated α-methyl or methyl carbons to form DNA adducts to produce its toxic, mutagenic, and carcinogenic effects. What determines NNK genotoxicity is the relationship between the metabolic activation of cytochrome P450 enzymes and constitutive DNA repair mechanisms [44]. Methane diazohydroxides and/or methyl diazo ions produced by the α-methylene hydroxylation of NNK react with DNA to produce mainly 7-N-methyl guanine (7-mGua) and O^6^-methyl guanine (O^6^-mGua) and a small amount of O^4^-methyl thymine. In contrast, α-methyl hydroxylation of NNK produces α-hydroxy methyl NNK, which is stable enough to undergo glucuronidation. It loses formaldehyde spontaneously to produce pyridoxabutazole hydroxide, which reacts with DNA to produce bulky pyridoxabutyration (POB) adducts [41].

It has been shown that NNK forms mainly indoors, especially at high nitric acid concentrations, but is reactive with O_3_ and NO, which can lead to its destruction, producing the major metabolite NNAL. NNK acts on the lung at relatively high concentrations, inducing mainly adenomas and adenocarcinomas [45] and is the only known pancreatic carcinogen in cigarette smoke and a biomarker of cardiovascular disease caused by tobacco smoke [36,42,46]. NNAL is the major carcinogenic metabolite of NNK and a dual-use biomarker of exposure to e-cigarettes and combustible cigarettes. Five pathways are known for the conversion of NNK to NNAL: carbonyl reduction, pyridine oxidation, α-hydroxylation (hydroxylation of the carbon adjacent to the N-nitroso group), denitroso reaction, and formation of ADP adducts. Carbonyl reduction of NNK is the major metabolic pathway and the carbonyl reductases involved in this process include 11-β-hydroxysteroid dehydrogenase (EC 1.1.1.146). This is a microsomal enzyme responsible for the interconversion of active 11-hydroxyglucocorticoid with the inactive 11-oxo form [44]. The metabolic transformation mode and oncogenic activity of NNAL are similar to those of NNK [39]. It has also been shown that in the absence of metabolic activation, NNK can be re-released from THS [39] trapped on the surface of an object to be activated by UV light and produce secondary contamination again [47].

Similar to NNK, NNN is classified as a class I carcinogen by the WHO and is found at the highest levels in tobacco and tobacco smoke. The concentration of NNN in cigarette smoke is higher than any other esophageal cancer carcinogen, so it can be used as a specific predictor of esophageal cancer risk in smokers [9]. Compared with NNK, NNN is more correlated with HONO, so the concentration of NNN increases as the concentration of HONO increases. That is, when NO_2_, a primary pollutant, increases in the indoor environment, HONO, a secondary pollutant produced by the reaction of NO_2_, also increases, and NNN is more susceptible to the relevant reactions. There are three main types of metabolism known for NNN: pyridine N-oxidation, hydroxylation of the pyrrolidine ring (including α-hydroxylation at the 2′- and 5′-positions and β-hydroxylation at the 3′- and 4′-positions), and norcotinine formation. Cytochrome P450 enzymes (CYP450s) mediate the 2′- and 5′-α-hydroxylation pathways, the major pathways of NNN metabolism, leading to the formation of DNA adducts [42,48]. Subsequently, the intermediate product of hydroxylation undergoes spontaneous decomposition to generate diazohydrides that can be further converted to alkyl diazo ions, which subsequently attack DNA and form various DNA damages such as POB-DNA adducts and pyridine-N-pyrrolidinyl (py-py)-DNA adducts. These damages can lead to tumorigenesis [48]. Detoxification pathways for NNN include the production of norcotinine, β-hydroxy NNNs, and NNN-N-oxide.

Unlike NNK and NNN, which have been widely described, NNA is a new and important chemical found in THS that is not present in fresh SHS and is the primary TSNA formed in THS when nicotine reacts with HONO (common indoor air pollution produced by direct emissions from indoor combustion devices and smoking, as well as the surface conversion of NO_2_ and NO) long after smoking has occurred [9]. The NNA content in THS exposed to HONO is at least three times greater than that of untreated HONO [9,48]. NNA can disrupt and break DNA strands and induce oxidative damage to the hypoxanthine phosphoribosyl transferase 1 (HPRT1) gene and the DNA polymerase beta (DNA polβ) gene [49]. Several experiments have shown that hepatocellular carcinoma cells exposed to NNA display high levels of DNA damage, and when damage levels are too high, the DNA polβ genes involved in DNA repair are over-regulated and induce major genetic changes associated with the malignant phenotype [50]. HPRT1 plays a key role in nucleotide metabolism [51], and its absence leads to dopamine deficiency and a decrease in 5-hydroxytryptamine receptors, which in turn triggers symptoms of uric acid overload in individuals, leading to lithiasis and arthrolithiasis, and even Lesch–Nyhan syndrome [51]. Iso-NNAL is another form of NNA, but it does not have tumorigenic activity. NNA is also as genotoxic as the carcinogen NNK at nanomolar levels of exposure [48].

Nicotinic acetylcholine receptors (nAChRs) polymorphisms are associated with lung carcinogenesis, and genotoxicity and a tumor-promoting environment are two necessary conditions for TSNAs to induce cancer. In addition to genotoxic effects, TSNAs linked to nAChRs expressed on the plasma membrane can affect lung cells. Acetylcholine (ACh) and its receptors play an extremely important role in both physiological functions and pathological processes of the body, mediating a variety of physiological processes, including muscle contraction, neurotransmission, and sensory transmission, by acting on nAChRs. These receptors are also crucial for tobacco addiction [52]since the addictive effects of nicotine are closely related to the rewarding effects mediated by nAChRs [53]. Cigarette smoking elevates nAChRs levels in the brain, and nicotine promotes nAChRs function and elevates post-transcriptional levels of nAChRs, promoting the formation of nAChRs pentamers and surface expression of receptors. There are various structures of nAChRs, though a pentameric structure consisting of five subunits is the most common, and each subunit contains an N-terminal extracellular structural domain for ligand binding, followed by four transmembrane (TM) regions. Between TM3 and TM4 is a large cytoplasmic region consisting of two structured helices, MX and MA, that regulate the interaction with cytoskeleton-anchored proteins. The common nAChRs subunit types are α7, α4β2, α6β2β3, α3β2, α6β4, and α9α10.

Nicotine binds to α7nAChRs present in neurons and α4β2nAChRs in dopamine neurons by mimicking acetylcholine. However, nicotine has a higher affinity for α4β2nAChRs [54]. The function of α7nAChRs, a regulator that stimulates cancer cells, was upregulated in smokers, whereas the function of α4β2nAChRs, which mainly regulates an inhibitory effect in tumors, was impaired [55]. Thus, the biological function of α7nAChRs is increased in smokers, whereas the function of α4β2nAChRs is impaired. nACHO is a neuron-specific endoplasmic reticulum-resident protein. As a specific molecular chaperone for neuronal nAChRs, nACHO is involved in α7 subunit folding, assembly, and cytosolic transport of α7nAChRs mainly through synergistic N-oligosaccharide transferase (OST), calmodulin, RIC3, and anti-apoptotic Bcl-2 protein [56]. nACHO is also involved in the biogenesis and function of α4β2 nAChRs. SAT1, the rate-limiting enzyme for polyamine catabolism metabolism, enhances α4β2 surface transport.

NNN and NNK are structurally similar to nicotine, though the affinity of NNN for the isomer αβnAChRs and NNK for α7nAChRs are 5000 and 1300 times higher than that of nicotine, respectively [57]. Through the action of α7nAchRs, NNA inhibits the activation of ERK1/2, which reduces the level of p-MAPK and affects cellular function. NNA, in turn, is affected by α3β2nAChRs in addition to α7nAChRs. The α3β2nAChRs act mainly in the sympathetic terminals around the cerebral vasculature, and nicotine is able to induce neurogenic vasodilation in the porcine basilar artery via α3β2nAChRs [58]. However, there is no direct evidence for the mechanism of action for NNA and α7nAchRs and α3β2-nAChRs.

The binding of NNK, a site-selective high-affinity agonist of α7nAChRs, to α7nAChRs activated voltage-gated Ca^2+^ channels, causing Ca^2+^ influx and membrane depolarization in lung cells. Subsequently, NNK activated the Raf-1/MAP kinase pathway by stimulating the release of the autocrine growth factor 5-hydroxytryptamine, leading to phosphorylation of c-myc, thereby regulating the growth of an important subset of small cell lung cancer (SCLC) and pulmonary neuroendocrine cells (PNECs) [59]. Stimulation of nAChRs with NNK leads to activation of three signal transduction effectors (GATA-3, nuclear factor-kappaB, and STAT-1), whereas NNN activates mainly GATA-3 and STAT-1. NNK- and NNN-induced GATA-3 protein binding activity was associated with elevated gene expression [60].

NNK, a β-adrenergic (β-ARs) agonist, is capable of stimulating DNA synthesis and proliferation of human pancreatic duct epithelial cells lung adenocarcinoma cells via the β-ARs release of arachidonic acid or trans-activation of epidermal growth factor receptor (EGFR) by initiating cAMP signaling [61,62]. The inhibitory neurotransmitter γ-aminobutyric acid (GABA) inhibits the β-ADR-initiated cAMP signaling cascade at the adenylyl cyclase level, blocking DNA synthesis and cell migration. The release of GABA requires the control of α4β2nAChRs, which is impaired in tobacco-exposed individuals [63,64]. In other words, NNK not only initiates the cAMP signaling pathway, it perpetuates its continuous activity, and eventually, cell migration and invasion occur. Adenocarcinoma has the highest correlation with smoking of all types of lung cancer [65], and smokers with chronic obstructive pulmonary diseases (COPD) are more likely to develop SCLC [66]. COPD is an inflammatory lung disease characterized by reduced exhalation of carbon dioxide and upregulated α7nAChRs levels [67]. In high CO_2_ and low O_2_ environments, NNK binds to α7nAChRs rather than β-ARs in healthy lungs [62,68,69]. Therefore, the risk of developing SCLC in patients with COPD will not be reduced in the absence of smoking if they are exposed to THS in an environment where smokers have lived for a long time.

A related study noted that an increase in the level of 8-hydroxy-2’-deoxyguanosine (8-OHdG) adducts, a marker of oxidative DNA damage, was detected in lung tissue after NNK injection, suggesting the ability of NNK to induce oxidative stress [70,71,72]. NNK mediates the onset of ROS, which not only allows DNA lesioning, but ROS act as signaling intermediates for many normal as well as pathological cellular processes to alter the microenvironment for tumorigenesis in vivo. NNA, which is unique to THS, can also induce DNA damage. NNA exposure reduces ribosomal protein S3 (RPS3), so less RPS3 translocates to the nucleus to bind to the 8-oxo-7,8-dihydroguanosine (8-oxoGuo) site of DNA lesions, and repair of 8-oxoGuo lesions is not activated; therefore, 8-OHdG will accumulate and DNA damage will increase. It was shown that the expression of the anti-apoptotic gene Bcl-2 was downregulated in cells under prolonged NNA exposure, and NNA was able to induce the expression of apoptosis-related genes [73]. At the same time, NNA was also able to disrupt the DNA repair system by decreasing the expression of RPS3, causing oocytes in the germinal vesicle stage to stop developing [74,75]. Furthermore, NNA exposure leads to an increase in mitochondrial Ca^2+^ ([Ca^2+^]m), resulting in abnormal mitochondrial distribution, dysfunction, accelerated ROS accumulation, and induced apoptosis [73].

**Figure 2:**
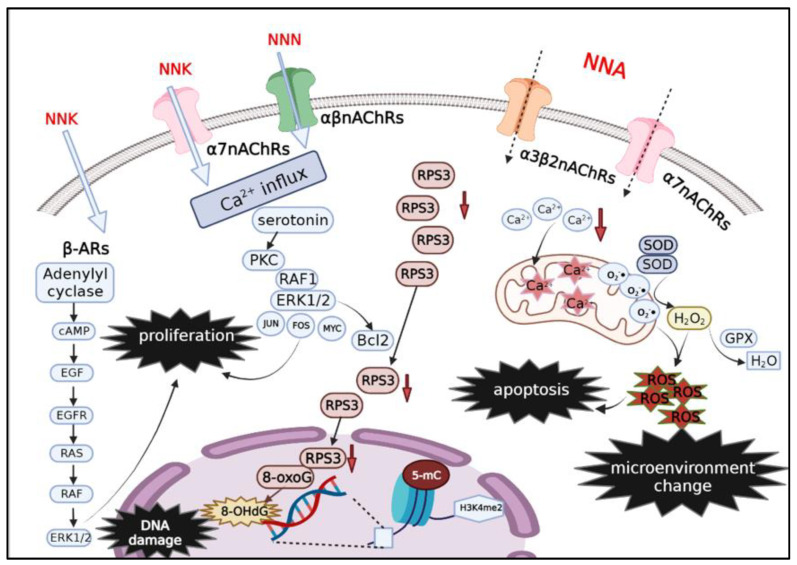
The mechanism of carcinogenesis of TSNAs (NNK, NNN, NNA) from THS. TSNAs can cause mutations in oncogenes and tumor suppressor genes, which affect cell proliferation, survival, migration, and invasion, and ultimately lead to cancer development. Metabolically activated TSNAs induce DNA adducts that can be eliminated by a functional DNA repair network, but unresolved DNA adducts further lead to mutations in oncogenes and suppressor genes, the first step in TSNAs-induced carcinogenesis. After NNK exposure, the level of the 8-OHdG adduct in lung tissue increased, which is a marker of DNA oxidative damage. NNA exposure decreased RPS3, so less RPS3 is transferred to the nucleus to bind 8-oxoGuo DNA lesions, and the repair of 8-oxoGuo lesions is not activated. Therefore, 8-OHdG accumulates, as does DNA damage. More interestingly, the decrease in the level of 5-mC level after NNA exposure alters DNA methylation levels and affects epigenetic modification. In addition, the binding of NNK, NNN, and NNA to nAChRs promotes tumor growth by regulating cell proliferation, cell survival, cell migration, and cell invasion, which is the second step in inducing cancer. The binding of NNK to α7nAChRs and NNN to αβnAChRs activates the Ca^2+^ channel of the voltage gate, causing Ca^2+^ to flood into lung cells, leading to membrane depolarization. In turn, protein kinase C, serine/threonine kinases, RAF1, mitogen-activated extracellular signal-regulated kinases (ERK) 1 and ERK2, and transcription factors FOS, JUN, and MYC are activated, leading to cell proliferation. NNK acts as an agonist of β-ARs and binds directly to them with high affinity to activate epidermal growth factor receptor (EGFR) via cAMP signals initiated by β-ARs, thus initiating the Ras/Raf/MEK/ERK-MAPK pathway and affecting cell proliferation. NNA may increase the level of mitochondrial Ca^2+^ and intracellular ROS by binding to α3β2nAChRs and α7nAChRs, thus affecting the cellular microenvironment.

More interestingly and specifically, NNA exposure altered epigenetic modifications in a way that other TSNAs did not. A decrease in 5-mC levels and a marked change in the level of DNA methyltransferase DNMT31 after NNA exposure suggested that NNA altered DNA methylation in oocytes; in addition, H3K4me2 levels were decreased and the methylation status of histones was altered (NNA was able to modify 2′-deoxyguanosine (dGuo), generating adducts including 8-Oxo-7,8-dihydro-2′-deoxyguanosine (8-oxo-dGuo), O^6^-methyl-dGuo, and N^2^-methyl-dGuo. Of these, O^6^- and N^2^-methyl-dGuo, are the first two methylated dGuo adducts to be identified. From the reaction between NNA and dGuo, a novel DNA glycan damage, 5′ and 3′-Methyl-dGuo, was also identified, which could lead to DNA backbone breakage if formed in the cell [76] (Figure 2).

## 6. Health Effects of THS

In summary, we know that THS, a secondary indoor pollutant, contains many carcinogenic substances. The accumulation of these carcinogenic components can have an impact on our health. We are already familiar with the health effects of active smoking and SHS. The first victim of passive smoking is the active smoker himself [77]. After smoking, nicotine remains on the smoker’s fingers and clothing and spreads as the smoker moves around. Many carcinogenic and toxic chemicals may be present at higher concentrations in SHS than the smoke inhaled by smokers. SHS can cause lung cancer, heart disease, and acute respiratory effects [78] and children exposed to SHS have an increased risk for acute respiratory infections, ear problems, and more severe asthma. In the fetal brain, nicotine activates nicotinic receptors, which play an important role in brain development [51]. Nicotine may be toxic to the developing brain by activating fetal nicotinic receptors that prematurely stimulate neuronal differentiation and exert cholinergic effects on cellular communication as well as mitosis [79]. In the previous section, we were able to show that THS contains as many potent carcinogens as SHS, and Ramírez et al. demonstrated that carcinogenic N-nitrosamines and TSNAs are widely present in smoking and smoke-free environments after measuring indoor dust samples from the homes of smokers and non-smokers [13]. Since THS resides on surfaces and in dust, chemical reactions with this residue can produce additional toxins. In other words, THS is a ubiquitous toxic substance, and its health effects cannot be ignored.

The initiation and progression of tumorigenesis are complex, involving the inactivation of tumor suppressor genes, activation of oncogenes and inflammatory processes, and alterations in the tissue microenvironment. Mistakenly repaired or unrepaired DNA adducts constitute another necessary step for the induction of cancer. THS causes genetic mutations in human cells, which contribute to the possibility of cancer and other diseases. THS extracts and NNA itself are genotoxic to human cell lines. Some studies have found that THS causes DNA gene strand breaks, which can produce unforeseeable serious consequences. A key event in the early stages of tobacco carcinogenesis is mutagenic DNA damage caused by genotoxic compounds, and NNK and NNN cause DNA damage and mutations through receptor-mediated actions and promote tumor growth, thereby inducing cancer [37]. In addition to the nitrosamines such as NNN, NNK and NNA described above, nicotine is oxidized in the presence of O_3_ and HONO to produce formaldehyde, N-dimethylformamide, and nicotinaldehyde, all of which are potential pulmonary toxins. THS exposure is also associated with increased oxidative stress. The binding of NNK and NNN to nAChRs enhances or deregulates cell proliferation, survival, migration, and invasion through the persistence of DNA adducts, such as those formed by the tobacco carcinogens PAHs and N-nitrosamines, which play a central role in tobacco-induced carcinogenesis. Furthermore, NNA is maternally toxic and impairs spermatogenesis, oocyte maturation, follicular development, and early development in F1 mice [73].

Martins-Green et al. found that exposure to THS caused significant damage to liver, lung, and healing skin in mice [80]. THS exposure stimulates the accumulation of adipocytes in the liver, leading to a significant increase in triglyceride and low-density lipoprotein (LDL) levels, a decrease in high-density lipoprotein (HDL), and defects in insulin metabolism. These metabolic changes predispose “metabolic syndrome” [80], which not only decreases the efficiency of oxygen diffusion, but also increases the risk of pulmonary fibrosis, which may lead to COPD and asthma. As with the consequences of active smoking, THS may also lead to poor wound healing in the skin due to a decrease in protofibrillar collagen in the tissues. Experiments have shown that rats exposed to THS are very active and develop hyperactivity [80]; there is also evidence that women exposed to tobacco prenatally increase the risk of their children developing attention deficit and hyperactivity disorder (ADHD) and behavioral disorders [81]. This suggests that the components of THS have a neurological effect in addition to direct effects on the body and maternal toxicity. In particular, infants and young children spend more time in closed environments and therefore may inhale dust particles contaminated with THS compounds [82]. Thus, THS may be more harmful to infants and children, and long-term exposure to tobacco may seriously affect their development. Children in environments where smoking is or has been permitted are at high risk of suffering multiple short- and long-term health problems, many of which may not fully manifest until later in life. Placental cadmium levels were higher in women who smoked compared to those who did not smoke [83] and blood cadmium levels were 3–4 times higher in smokers than non-smokers. Matt et al. examined the composition of settled house dust and found that the cadmium and lead components of THS persisted long after smoking ended [84]. Cadmium in cigarette smoke is known to be a possible cause of lung cancer in smokers, and exposure to lead and cadmium in THS may lead to cardiovascular disease, kidney disease, and osteoporosis [85]. THS is a major threat not only to human health but also to household pets. Pet cats are inevitably exposed to THS on floors, furniture, and household items, which not only increases the risk of lymphoma in pet cats but may also induce squamous epithelial cell carcinoma in the mouth and even tumors in the nasal cavity. Gastrointestinal disorders and skin allergies in cats are also often associated with smoking environments [86].

## 7. THS Detection

The most common method currently used to detect THS contamination is surface wipe sampling to analyze environmental concentrations of nicotine and cotinine. Other commonly used markers include cotinine TSNAs such as NNK and PAHs. Cotinine is both an alkaloid in tobacco and a major metabolite of nicotine, with a longer half-life than nicotine (10–40 h). This method is fast, simple, and accurately reflects the concentration of nicotine in indoor air. Environmental detection of THS often uses a combination of two or more markers, such as nicotine and 3-vinylpyridine, nicotine and TSNAs, or one of the main tobacco-specific pyridine-containing compounds can also be used. By using multiple markers and measuring their ratios, the growth and decline of different contaminants over time can be better assessed.

## 8. Biomarkers of THS

Identifying biomarkers of tobacco chemicals is the key to assessing the health effects associated with THS exposure. A common way to assess the type, extent, and frequency of tobacco smoke exposure is biomonitoring the major nicotine biomarker cotinine in urine, blood, and saliva. Biomarkers are subdivided into exposure markers and effect markers, and the priority factor is their specificity. Tobacco-specific biomarkers—nicotine and TSNAs—are chemicals extracted from tobacco smoke. Compared to SHS, THS exposure has several different characteristics. First, because TSNA concentrations accumulate over time, non-smokers exposed to THS typically have much higher TSNAs/cotinine ratios than non-exposed individuals, and there is no gender, racial/ethnic, or age differences [87,88]. This ratio can therefore be used as a biomarker to distinguish between SHS and THS exposure. Furthermore, NNA, as a major product in THS and not present in fresh SHS, or NNA-dG covalently bound adducts could serve as such a biomarker to identify individuals exposed to THS, thus providing important information for early detection and prevention [76]. An assessment of the possible major metabolites of NNA, iso-NNAL, and 4-(methylnitrosamine)-4-(3-pyridyl)butanoic acid (iso-NNAC) is also available to distinguish between second-hand and THS exposure. Pratt showed that the levels of biomarkers such as NNK and cotinine increased significantly with THS exposure, confirming the reliability of using these biomarkers for screening for THS exposure [89]. In addition, Jacob et al. detected nicotelline in aged house dust smoke particles and were able to detect metabolites of nicotelline in the eyes of smokers and birds, so it may be a new biomarker for THS [90]. However, biomonitoring studies of these specific NNA biomarkers have been lacking to date. In addition, the main routes of exposure to THS, compared to SHS, are non-dietary intake and dermal absorption. Non-smokers are exposed to smoke toxins attached to fabrics, clothing, dust, and surfaces in an environment where THS is present. During this process, the skin functions as an effective barrier, but nicotine can be absorbed by the skin and transported to the cutaneous blood supply [91]. Different studies have shown that the skin penetration factor of nicotine is large and that absorption of nicotine by the skin can occur directly from the air, which is comparable to the estimated absorption of inhaled nicotine [91,92]. Therefore, biomonitoring of toxicants accumulated in the skin is one of the priorities in the selection of biometrics for marker detection and the detection of biomarkers of THS exposure. As THS exposure is similar to SHS exposure, it can lead to the absorption of complex mixtures of toxic substances. Concentrations of biomarkers of tobacco exposure will vary depending on the predominance of the source of exposure (SHS or THS), so a more comprehensive assessment using multiple biomarkers will help us to better understand its impact on human health [93].

## 9. Conclusions

The presence of THS as an indoor secondary pollutant is a newly identified health risk. Residual indoor nicotine reacts with a common indoor pollutant, HONO, to form mutagenic TSNAs. Although NNK and NNN are present in both SHS and THS, NNA is only present in THS. NNK and NNN have been extensively studied as human carcinogens. However, relatively little information is available on the genotoxicity and DNA reactivity of NNA, the main product of THS. NNN, NNK, and NNA all affect cell activity by producing DNA adducts and affecting DNA and mitochondrial DNA strand break and oxidative damage, or by promoting the tumor growth microenvironment by binding to nAChRs and regulating the regulation of cell proliferation, survival, migration, and invasion. It is worth mentioning that NNA can also affect gene expression through epigenetic modalities. Most of the findings suggest potential health effects from THS exposure (i.e., alterations in cytotoxicity, metabolism, blood glucose, or cell structure; alterations in liver, lung, skin, and behavior in mice) and a lack of awareness of the risks of THS in the general population. The primary focus of current research trends in THS is to obtain more evidence on the biological and human health risks of THS, which is fundamental to scientific research, public perception, and policy development. Furthermore, research and development of THS markers are urgently needed to facilitate pollution monitoring, health risk assessment, and early prevention of diseases related to THS environments. Many factors in the indoor environment can affect the concentration and distribution of THS and the nature of its components, and the components of THS may also react with other pollutants in the environment to produce other secondary pollutants.

Although we do not currently know exactly what the health risks of THS can be, as a reaction product of SHS, THS is theoretically harmful at any exposure dose. To date, there is also no way to eliminate THS. Different time applications and activity patterns place people in different environments throughout the day. Both indoors and outdoors, primary pollutants are released directly into the environment and the secondary pollutants that are generated through reactions are inevitably present in life and pose a continuous health hazard to living organisms. THS, as an indoor secondary pollutants, is ubiquitous as a long-term potential health threat. THS is difficult to isolate, even in non-smoking areas, and it is difficult to completely remove THS from indoors using ventilation. It is difficult to determine how long it is safe to enter a room where THS is present after ventilation, and measures such as cleaning the room and painting the walls may not completely solve the problem of THS. Although smokers should smoke outdoors, nicotine residue still adheres to the smoker’s skin or clothes, and it is unavoidable that it will still spread everywhere when the smoker returns indoors. Therefore, effective prevention of THS is very important. THS is ultimately man-made pollution caused by human smoking, so enhancing public awareness of THS is a priority that can help the public avoid or reduce their exposure to THS. The development and strict enforcement of public policies are key to preventing THS pollution.

## Figures and Tables

**Table 1:** The main components of SHS emissions and their representative substances.

Major Components	Vapor Pressure Range	Representative Compounds in the Components
Gas-phase inorganic compounds	>13 kPa	CO_2_, CO, NH_3_
Very volatile organic compounds(VVOCs)	>7 to 13 kPa	Formaldehyde, acrolein, 1,3-butadiene, acetylaldehyde
Volatile organic compounds(VOCs)	0.01 to 10 kPa	Benzene, styrene, toluene, 2-butanone, N, N-nitrosodimethylamine, N-nitrosopyrrolidine
Semi-volatile organic compounds(SVOCs)	10^−2^ to 10^−8^ kPa	Nicotine, N-nitrosonomicotine, 4-(methylnitrosamino), 1-(3-pyridyl)-1-butanone
Particulate organic compounds	<10^−8^ kPa	Benzo[α]pyrene, benzo[β]fluoranthene, benzo[κ]fluoranthene, solanesol

## Data Availability

Not applicable.
